# Localization of the Epileptogenic Zone by Multimodal Neuroimaging and High-Frequency Oscillation

**DOI:** 10.3389/fnhum.2021.677840

**Published:** 2021-06-08

**Authors:** Xiaonan Li, Tao Yu, Zhiwei Ren, Xueyuan Wang, Jiaqing Yan, Xin Chen, Xiaoming Yan, Wei Wang, Yue Xing, Xianchang Zhang, Herui Zhang, Horace H. Loh, Guojun Zhang, Xiaofeng Yang

**Affiliations:** ^1^Laboratory of Brain Disorders, Collaborative Innovation Center for Brain Disorders, Ministry of Science and Technology, Beijing Institute of Brain Disorders, Capital Medical University, Beijing, China; ^2^Xuanwu Hospital, Capital Medical University, Beijing, China; ^3^Bioland Laboratory, Guangzhou, China; ^4^College of Electrical and Control Engineering, North China University of Technology, Beijing, China; ^5^MR Collaboration, Siemens Healthcare Ltd., Beijing, China

**Keywords:** epileptogenic zone, neuroimaging, high-frequency oscillations, PET-MRI, FLAWS, multimodal method

## Abstract

Accurate localization of the epileptogenic zone (EZ) is a key factor to obtain good surgical outcome for refractory epilepsy patients. However, no technique, so far, can precisely locate the EZ, and there are barely any reports on the combined application of multiple technologies to improve the localization accuracy of the EZ. In this study, we aimed to explore the use of a multimodal method combining PET-MRI, fluid and white matter suppression (FLAWS)—a novel MRI sequence, and high-frequency oscillation (HFO) automated analysis to delineate EZ. We retrospectively collected 15 patients with refractory epilepsy who underwent surgery and used the above three methods to detect abnormal brain areas of all patients. We compared the PET-MRI, FLAWS, and HFO results with traditional methods to evaluate their diagnostic value. The sensitivities, specificities of locating the EZ, and marking extent removed versus not removed [RatioChann(ev)] of each method were compared with surgical outcome. We also tested the possibility of using different combinations to locate the EZ. The marked areas in every patient established using each method were also compared to determine the correlations among the three methods. The results showed that PET-MRI, FLAWS, and HFOs can provide more information about potential epileptic areas than traditional methods. When detecting the EZs, the sensitivities of PET-MRI, FLAWS, and HFOs were 68.75, 53.85, and 87.50%, and the specificities were 80.00, 33.33, and 100.00%. The RatioChann(ev) of HFO-marked contacts was significantly higher in patients with good outcome than those with poor outcome (*p*< 0.05). When intracranial electrodes covered all the abnormal areas indicated by neuroimaging with the overlapping EZs being completely removed referred to HFO analysis, patients could reach seizure-free (*p* < 0.01). The periphery of the lesion marked by neuroimaging may be epileptic, but not every lesion contributes to seizures. Therefore, approaches in multimodality can detect EZ more accurately, and HFO analysis may help in defining real epileptic areas that may be missed in the neuroimaging results. The implantation of intracranial electrodes guided by non-invasive PET-MRI and FLAWS findings as well as HFO analysis would be an optimized multimodal approach for locating EZ.

## Introduction

For patients with drug-refractory epilepsy, surgical removal of the epileptogenic zone (EZ), which is defined as the brain regions necessary and sufficient for initiating seizures (Rosenow and Luders, 2001), is the best option to eliminate seizures (Jacobs et al., 2010). Because the definition of the EZ is only a theoretical concept, as of now, there are no techniques that can accurately delineate an EZ. Recently, some new techniques, which could help in identifying the EZs have attracted increasing attention, including PET-MRI (positron emission tomography-magnetic resonance imaging), special MRI sequences, a neuroimaging postprocessing technique, and an automatic analysis of high-frequency oscillations (HFOs) (Salamon et al., 2008; Holler et al., 2015; Soares et al., 2016; Sun et al., 2018).

PET imaging provides important insights into the functional integrity and activity of neural systems, as it is a sensitive and non-invasive method for measuring brain metabolism. However, PET imaging has limitations of a lower spatial relationship and false lateralization (Chugani et al., 1993; Hur et al., 2013). Newly developed PET and MRI coregistration approaches combined the tissue contrast of MRI and the metabolic characterization of FDG-PET, which would help to delineate surgical resection margins correlated with the structural anatomy and improve surgical outcomes based on discernible MRI findings (Salamon et al., 2008). In addition, hybrid PET/MR scanners could combine the superior soft tissue contrast of MRI and the metabolic characterization of FDG-PET into a single exam without the need for the additional ionizing radiation inherent to PET/CT systems (Paldino et al., 2017), thus, further promoting its clinical usage.

In the analysis of MRI results, the method of separating gray matter (GM), white matter (WM), and cerebrospinal fluid (CSF) in the brain has been very important in a variety of clinical studies and neuroscience research fields, as it enables the quantification of tissue volume and visualization of anatomical structures (Grieve et al., 2013; Erickson et al., 2014; Gautam et al., 2014; Schick, 2016). Recently, a novel sequence known as fluid and white matter suppression (FLAWS) was developed to obtain three different anatomical images with high spatial resolution in one scan (Tanner et al., 2012). It first acquires two sets of 3D images using two inversion times, with TI1 suppressing the WM signal and TI2 suppressing the CSF signal. Based on these two sets of images, a set of synthetic minimum FLAWS-contrast images is subsequently calculated, which suppresses both the WM and CSF signals (gray matter specific). Gray matter-specific contrast images have been reported to detect abnormal brain structures more accurately than do conventional MRIs (Wang et al., 2018), which is important for epilepsy surgery.

Over the past 20 years, several studies reported an association between the removal of areas showing high rates of HFOs and a good surgical outcome (Jacobs et al., 2010; Akiyama et al., 2011; Fujiwara et al., 2016; Frauscher et al., 2017; Jiang et al., 2018; Ren et al., 2018), suggesting that HFOs are a promising biomarker of epileptogenic tissues. HFOs are generally viewed as spontaneous EEG patterns at frequencies ranging from 80 to 500 Hz. They consist of at least four oscillations that are clearly differentiated from the background activity, classified into ripples (80–200 Hz) and fast ripples (FRs; 200–500 Hz).

No technique has yet been able to precisely locate the EZ, and a few reports have described the application of multiple technologies to form a multimodal method with improved accuracy in localizing the EZ. Therefore, in this study, we aim to retrospectively explore the possibility of using a multimodal method that combines PET-MRI, FLAWS, and an automated analysis of HFOs to delineate the EZ. We also aimed to determine the accuracy of different combinations of technologies in locating the EZ.

## Materials and Methods

### Patient Selection

In this study, 55 patients were consecutively enrolled from March 2016 to July 2019. All patients underwent long-term intracranial electroencephalogram (iEEG) monitoring. The inclusion criteria were as follows: (1) the patient underwent at least two of the following three examinations: PET-MRI, FLAWS, and HFOs; (2) the epileptogenic region was resected or ablated; and (3) at least 12 months of postsurgical follow-up was completed.

This study was approved by the ethics committee of Xuanwu Hospital of Capital Medical University, and all patients have signed the informed consent form.

### Positron Emission Tomography-Magnetic Resonance Imaging Scanning and Image Processing

#### Positron Emission Tomography and Magnetic Resonance Imaging Infusion Image Collection

Some patients underwent a regular MRI scan of the brain using a MAGNETOM Trio Tim 3T MR scanner (Siemens Healthcare, Erlangen, Germany) and received [^18^F] FDG PET-CT scans to extract the PET signal from the PET-CT images for imaging registration. Structural images were acquired with a three-dimensional T1-weighted sequence (TR = 1,600 ms, TE = 2.15 ms, flip angle = 9°, thickness = 1.0 mm, and FOV = 256 mm × 256 mm). The registration was conducted using SPM12 software^1^.

#### Hybrid Positron Emission Tomography-Magnetic Resonance Imaging Scan

Some patients received a hybrid PET-MRI scan, which simultaneously obtains higher resolution PET images and MRI images. The process was performed on an integrated simultaneous Signa PET-MRI imaging system (GE Healthcare, Milwaukee, WI, United States). The MRI portion of the hybrid PET-MRI was acquired with a 3.0-T MRI using a 19-channel head coil that obtained all sequences of MRI, including the BRAVO sequence that is the same as MPR images. Parameters for the routine anatomic acquisitions were as follows: axial T2-weighted fast spin-echo (TR = 9,600 ms, TE = 149 ms, matrix size = 256 × 256, slice thickness = 3.0 mm, and gap = 1.0 mm); axial T1-weighted fast spin-echo (TR = 3,286 ms, TE = 24 ms, matrix size = 288 × 256, and slice thickness = 3.0 mm, gap = 1.0 mm). The PET portion of the hybrid PET-MRI is based on the magnetic compatibility of the good solid phase array-type optoelectronic digital converter (Silicon Photomultiplier, SiPM) of the PET detector, using time-of-flight (TOF) technology. Digital MRI information and PET image information, together with TOF technology, significantly improve the accuracy and precision of MR-based attenuation correction (MRAC), and sequentially, accurately, and quantitatively display lesions associated with epilepsy. The acquisition protocol and other detailed parameters can be obtained from previous publications (Shang et al., 2018).

#### Imaging Review

Two experienced radiologists who were blinded to the location of the resected regions separately reviewed the images and marked abnormal areas showing obvious hypometabolism. Differences in assessments between the radiologists were resolved by consensus.

### Fluid and White Matter Suppression Examination

FLAWS imaging was conducted on a 3T MAGNETOM Verio MRI scanner (Siemens Healthcare, Erlangen, Germany). The output of the FLAWS sequence consisted of two sets of images: the 3D volume acquired using the TI1 sequence (FLAWS1) and the volume acquired using the TI2 sequence (FLAWS2). Detailed parameters for FLAWS were TR = 5,000 ms, TE = 2.88 ms, TI1 = 409 ms, TI2 = 1,100 ms, flip angel = 5°, matrix size = 256 × 256, slice thickness = 1.0 mm, and FOV = 256 mm × 256 mm (Chen et al., 2018).

The images were reviewed by two radiologists, and the final conclusions have reached a consensus. Detailed standards could be found in the paper above.

### Recording and Analysis of High-Frequency Oscillation

#### Electrode Types and Intracranial Electroencephalogram Recordings

Subdural electrodes (contact diameter of 4 mm, 2.5-mm exposure, and 10-mm spacing between contact centers) and SEEG electrodes with 8, 10, 12, and 16 contacts (0.8-mm diameter, 2-mm length, and 1.5-mm between contacts; Beijing Huakehengsheng Healthcare Co., Ltd., Beijing, China) were implanted in the putative epileptogenic region. The iEEGs were acquired using a 256-channel Nicolet recording system (Natus Medical Incorporated, San Carlos, CA, United States) with a sampling rate of 4,096 Hz. At the same time, we recorded the submental electromyogram (EMG) and the electro-oculogram (EOG) to monitor the sleep stage.

#### Automatic Detection of High-Frequency Oscillations

We selected 5 min of a slow wave sleep epoch when the delta activity occupied more than 25% of background activity in 30-s epochs. We referred to the results of the EMG and EOG obtained from interictal periods that were separated by at least 2 h from seizures and were transformed to a bipolar montage composed of adjacent contacts.

For automated detection of ripples and FRs, we used the method developed by our team (Jiang et al., 2018; Ren et al., 2018). We used a zero-phase finite impulse response filter, and the cutoff frequencies were 80–200 and 200–500 Hz for ripples and FRs, respectively. As described in our previous paper, we plotted a peak point distribution curve (PPDC) by computing the absolute amplitude of all peak points in each channel and ranking them in ascending order. According to maximum distributed peak points algorithm, all peak points before the turning point of PPDC are baseline points. We assigned the mean baseline amplitude by calculating the mean peak point amplitude before the turning point with a 5-s moving window. Next, ripples and FRs were automatically detected when the corresponding frequency band of signals met our setting thresholds. We set ripples as oscillations with at least eight consecutive peaks higher than 3 standard deviations (SD) above baseline mean amplitude and six consecutive peaks higher than 10 SD above baseline mean amplitude; and FRs were set as oscillations with eight consecutive peaks higher than 3 SD above the baseline mean amplitude and six peaks higher than 9.5 SD above baseline mean amplitude. Then, we automatically removed false HFOs caused by Gibbs effect through computing the frequency offset power difference of phase space reconstruction. Only when the power difference was bigger than zero would the corresponding HFO be reserved for further analysis. Last, we ranked all channels in a descending order according to the HFO rates for each patient and the top 72% channel distribution area of FRs was considered as the EZ. When no FR was detected, we would refer to the ripples results.

### Outcomes of Seizures

The postsurgical outcomes of the patients were classified using the scheme described by Engel (Jerome Engel, 2016). We defined a good postsurgical outcome as class I (seizure-free) and a poor postsurgical outcome as class II and III (recurrent seizures).

### Classification of Electrode Contacts and Contacts Surgically Removed

Preimplantation MRI and post-implantation CT scans could help in locating each contact anatomically along the electrode trajectory. In addition, the postsurgical MRIs were used to determine whether electrode contacts were included in the brain tissue that was eventually removed during surgery.

### Single Comparison

#### Comparing Results Obtained by New Techniques With Those Obtained by Traditional Methods

As some traditional inspection methods like MRI, scalp EEG, and intracranial EEG are widely used clinically, it is necessary to compare their results pertaining to the detection of suspicious epileptic areas with those of new techniques such as PET-MRI, FLAWS, and HFOs, to evaluate the diagnostic value. Techniques with more accurate marking areas were considered to have higher diagnostic values.

#### Comparing the Efficiency of Locating EZ

Based on the surgical outcome, we selected two groups of patients (I—no seizure and II —few seizures). For patients with a good outcome, each patient’s brain area could be regarded as two parts: one part was the area marked by the method and eventually removed finally, which must belong to the EZ; the other part was the remaining area, which must be inside the non-EZ. For patients with few seizures and notable improvement, the removed marking area must be part of the EZ, and the other part must contain portions of the EZ. If the marking area was part of the EZ, it is considered true positive; otherwise, it is false positive. Because we could not define the marking areas that have not been removed as either epileptic or normal, patients with non-removing marking areas were excluded. Then we calculated the sensitivity and specificity of each method.

In addition, we also used RatioChann(ev) to evaluate their locating value. According to the PET-MRI, FLAWS, and HFO results, we separately marked the channels in the abnormal area. We calculated the ratio of the resected channels to the total channels in the abnormal areas marked using the three methods. Only patients whose implanted electrodes had covered all the abnormal areas identified using PET-MRI or FLAWS were included here. We named the ratio RatioChann(ev).

$$R\,a\,t\,i\,o\,C\,h\,a\,n\,n\,\left(e\,v\right)=\frac{\#\,C\,h\,a\,n\,n\,R\,e\,m\,\left(e\,v\right)-\#\,C\,h\,a\,n\,n\,N\,o\,n\,R\,e\,m\,\left(e\,v\right)}{\#\,C\,h\,a\,n\,n\,R\,e\,m\,\left(e\,v\right)+\#\,C\,h\,a\,n\,n\,N\,o\,n\,R\,e\,m\,\left(e\,v\right)}$$

where, ev is the type of event (PET-MRI, FLAWS, or HFOs-FRs), #ChannRem is the number of removed channels marked by events, and #ChannNonRem is the number of non-removed contacts with events (Jacobs et al., 2010; Gliske et al., 2018). We compared RatioChann(ev) with outcome.

### Multimodal Comparison

#### Comparing Abnormal Regions Marked Using the Three Methods

We compared the abnormal regions marked by PET-MRI, FLAWS, and HFOs in different combinations, and we calculated the consistencies of the different combinations. Because some factors (such as sex, onset age, onset duration, seizure frequency, and involved lobes) might affect the consistency, we also compared the consistencies in different conditions.

Furthermore, we analyzed the correlations between the overlapping areas identified using the three methods (at least two or more methods) with the removing area and outcome. We compared the correlations between completely and partially/not removing marking areas and outcome in the following four groups: (1) PET-MRI, (2) FLAWS, (3) HFOs, and (4) multimodally.

#### Comparison of the Extent of Areas Marked Using the Three Methods

We compared the extent of areas marked in each patient using the PET-MRI, FLAWS, and HFOs to ultimately investigate the correlations among the three methods. Because the lesions were different, we specifically selected patients with FCD confirmed by postsurgical pathology, for further comparison.

### Statistical Analysis

Sensitivity = true positive/(true positive + false negative); specificity = false positive/(false positive + true negative). A Wilcoxon rank sum test was used to compare ratios between different groups. Fisher’s exact test was used to test the correlation between complete removing and outcome. Statistical significance was established at p < 0.05.

All statistical analyses were performed using IBM SPSS Statistics 17 software (IBM Corp., Armonk, NY, United States).

## Results

### Patient Selection and Surgical Outcomes

A total of 55 patients, 15 were ultimately enrolled in this study, and 40 patients were excluded for not undergoing PET-MRI scan or FLAWS examination. Patient characteristics are summarized in Table 1. Ten patients had all the methods’ results, one patient had HFO and FLAWS results, and four patients had HFO and PET-MRI results. Notably, (1) among the 14 patients with available PET-MRI results, 7 patients underwent a hybrid PET-MRI scan, and the results for the other patients were derived from FDG-PET and MRI imaging coregistration; (2) Patient # 15 had no FRs detected by our detector, and the related HFO location information was given by ripples results. For the variable uniformity, the RatioChann(ev) comparisons related to FRs excluded this patient. One year after the operation, 10 patients experienced recurrent seizures after surgery (5 with Engel II, and 5 with Engel III).

**TABLE 1:** Clinical information for all patients.

Patient	Sex/Age	Onset/Fre	Electrode	PET-MRI	FLAWS	HFOs	Overlapped	Removing	Outcome
1	M/23	17/d	SEEG	LT,LPT,LMP	LT,LPT,LMP	LH,LPT	LH,LPT,LMP	LH, LPT	I
2	M/29	5/d	SEEG	RT,RH	RT, RH	RH,RA	RH,RT	RT, RH,RBF	I
3	F/42	12/m	SEEG	RSP	RP	RP, RIP	RP	RP	II
4	M/16	7/w	SEEG	RPT	Normal	RPT,LH	RPT	RPT	II
5	M/13	5/d	SEEG	RCS	RCS	RP, RCS	RCS	RCR(*)	II
6	F/17	1/w	SEEG	RT,RP	RF,RCR	RF,RCR	RF,RCR	RF	III
7	M/20	6/m	SEEG	LF,LOF,LTP,LH	LF,LOF,LT,LO,RO	LF,LH,RA	LF,LOF,LH	LOF,LTP,LH	III
8	F/26	9/w	SEEG	RF,RT,RI	RT,RI	RT	RTRI	RIO(*)	III
9	M/26	13/d	SEEG	RF	RF	RF	RF	RF	II
10	F/33	13/d	Sub/depth	LP,LI	LT	LO,LT,U	LT,LI	LT,LI	III
11	M/20	3/w	Sub/depth	/	RF,RCR	RF,RCR	RF,RCR	RF	III
12	F/26	5/w	SEEG	LF	/	LF	LF	LF	I
13	F/13	12/w	SEEG	LF	/	LF	LF	LF	I
14	F/41	19/m	SEEG	RF	/	RF,RMCC	RF	RF,RCR	II
15	M/16	15/d	SEEG	RF	/	RF(#)	RF	RF	I

LT, left temporal; LPT, left posterior temporal; LMP, left medial parietal; LH, left hippocampus; RT, right temporal; RH, right hippocampus; RA, right amygdala; RACC, right anterior cingulate cortex; RMCC, right middle cingulate cortex; RBF, right basis frontalis; RF, right frontal; RSP, right superior parietal; RP, right parietal; RIP, right inferior parietal; LH, left hippocampus; RCS, right central sulcus; RCR, right central region; LF, left frontal; LTP, left temporal pole; LOF, left orbitofrontal; LO, left occipital; RO, right occipital. FCD, focal cortical dysplasia. M, male; F, female; Fre, seizure frequency; d, having seizure every day; w, having seizure every week; m, having seizure every month; Sub, subdural electrode. *The patient has undergone radiofrequency ablation instead of resection. #No fast ripple (FR) was detected (the results were from ripples).

### Diagnostic Value of Single Method Compared With Traditional Ones

For the MRI scan, 7/15 (46.67%) patients had normal results, 1 patient had bilateral abnormalities, and 14/15 (93.33%) patients’ MRI results were not consistent with scalp EEG results. For the scalp EEG monitoring, one patient had normal conclusion, two patients had bilateral locations, and others seem to have too broad locations. The iEEG results seemed to be more local and accurate but still easily missed some potential abnormalities compared with HFO analysis.

Compared with the traditional methods, PET-MRI, FLAWS, and HFOs were able to locate more potential epileptic areas or to locate the abnormal areas more focally. This meant that the new methods could provide more useful information to clinicians, and further help in locating the EZ more effectively (Supplementary Table 1).

### Efficiency of Finding the EZs

When finding the EZs, we calculated that the sensitivity and specificity of PET-MRI were 68.75 and 80.00%, respectively. The sensitivity and specificity of FLAWS were 53.85 and 33.33%, respectively. The efficiency of HFOs seemed to be better, reaching 87.50% sensitivity and 100.00% specificity.

When comparing RatioChann(ev) of removal outcome, for PET-MRI and FLAWS, there were no significant differences in the ratios between patients with good and poor outcomes (*p* = 0.6954, *p* = 0.6667, Figure 2C). The ratio observed for removed and non-removed HFO-marked contacts was significantly higher in patients with a good outcome than in patients with a poor outcome (p = 0.0270, Figure 2C).

### Consistency of the Three Methods

**FIGURE 1:**
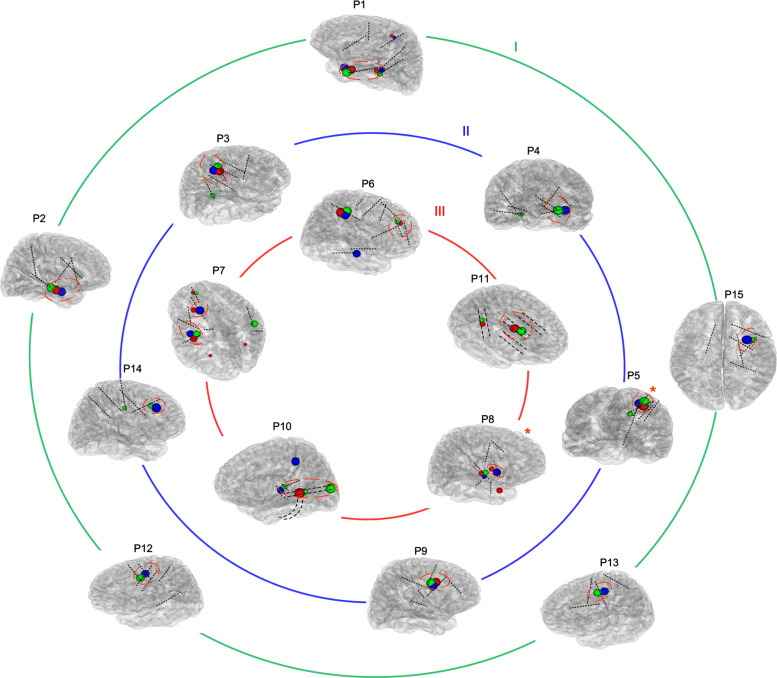
Reconstructions of the placement of intracranial electrodes and suspicious areas mapped in all patients. The images showed the locations of subdural electrodes (P10 and P11) or SEEG locations in all 15 patients. Most electrodes are shown in black, while one contralateral electrode that was unable to be shown in the brain model is presented in white. Blue, red, and green globes mark suspicious abnormalities detected using positron emission tomography-magnetic resonance imaging (PET-MRI), fluid and white matter suppression (FLAWS), and high-frequency oscillations (HFOs), respectively, and the different volumes indicate different levels of significance. The removed volume is also marked by a red imaginary line. Moreover, all patients were divided into three groups according to their outcomes (Engel I, II, and III), as shown by the three circles. *The patient has undergone radiofrequency ablation instead of resection.

**FIGURE 2:**
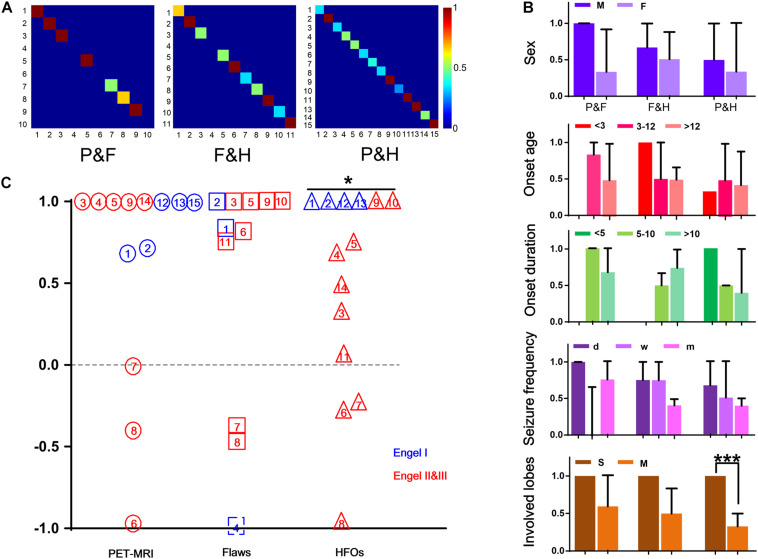
Consistency of the three methods. **(A)** The three images show the consistency of PET-MRI and FLAWS (P&F), FLAWS, and HFOs (F&H), PET-MRI and HFOs (P&H), and colors closer to red indicate better consistency. **(B)** The consistencies in different conditions are shown here. Different colors represent different groups (sex, onset age, onset duration, seizure frequency, and involved lobe). *** *p* = 0.001. **(C)** RatioChann(ev) for each patient obtained using the three methods are listed in this figure. PET-MRI, FLAWS, and HFOs are listed from left to right and are indicated by circles, boxes, and triangles, respectively. The numbers written in the different patterns indicate different patients. Additionally, patients’ outcomes are marked in different colors. For patient #4, the FLAWS examination did not identify an abnormality, and the ratio of removal was unable to be determined; therefore, we marked it with an imaginary line. For patient #10, electrodes were not implanted in all suspicious areas marked by PET-MRI, and thus, the patient was excluded from the comparison. *p < 0.05.

Of the 14 patients who underwent PET-MRI scans, at least one of the abnormal brain regions detected using PET-MRI showed a high rate of HFOs (14/14, 100%). In 5 of these 14 patients, PET-MRI and HFOs showed exactly the same location of abnormal areas (5/14, 35.71%). In 10 of the 11 patients who had FLAWS data, at least one of the abnormal areas identified using FLAWS showed a high rate of HFOs (10/11, 90.91%); moreover, the EZ defined that using the two aforementioned methods was completely consistent in 4 of the 11 patients (4/11, 36.36%). Patients, 9 of 10, who underwent both a PET-MRI scan and a FLAWS examination had at least one matched abnormal area (9/10, 90.00%), and the suspicious areas were completely consistent in 5 patients (5/10, 50.00%) (Figures 1, 2A).

By comparing the different factors (sex, onset age, onset duration, seizure frequency, and involved lobes) among patients with consistency in results from different methods, we observed a tendency toward better consistency between PET-MRI and HFOs in patients who had single involved lobes (*p* = 0.001) (Figure 2B).

In all (100%) the patients, PET-MRI and/or FLAWS could find abnormal areas. Among them, two patients’ intracranial electrodes did not cover all the suspicious areas. Thus, the relative abnormal discharge could not be captured, which might contribute to their Engel III outcome. Three other patients also developed Engel III outcome due to the incomplete removal of overlapping areas marked by at least two methods. For 10 patients whose electrodes covered all suspicious zones indicated by neuroimaging, indicating a complete removal, better outcome was detected after 1 year, and 5 with few seizures had residual FRs areas (Figure 1).

### Correlation With Outcome Individually or Multimodally

When using the method individually, we found that only patients in whom the HFO-marked areas were completely resected had larger chance of seizure freedom compared with partial groups (*p* = 0.007).

Considering the results shown above, we define the multimodal group as: 1) whether intracranial electrodes covered all suspicious areas indicated by neuroimaging and 2) whether the HFO-marked areas were completely removed. The results showed that the multimodal group had a significantly higher possibility of being seizure-free when both conditions were met (*p* = 0.002) (Table 2).

**TABLE 2:** Correlation with outcome individually or multimodally.

	Total	Seizure-free	Non-seizure-free	*P* value
**PET-MRI areas completely removed?**				
Yes	10	4	6	1
No	4	1	3	
**FLAWS areas completely removed?**				
Yes	6	1	5	1
No	5	1	4	
**HFOs areas completely removed?**				
Yes	7	5	2	0.007 (**)
No	8	0	8	
**(1) Electrodes covering all neuroimaging areas; (2) Completely removing HFOs areas?**				
Yes	6	5	1	0.002 (**)
No	9	0	9	

**p < 0.01.

### Comparison of the Extent of Marking

After comparing the extent of areas in each patient marked using the three methods, in most cases, PET-MRI covered the largest abnormal (hypometabolism) areas. FLAWS marked relatively smaller abnormal areas. HFOs marked the most focal meaningful areas, which were located in the core or the margin of the lesion or the perilesion marked by neuroimaging.

**FIGURE 3:**
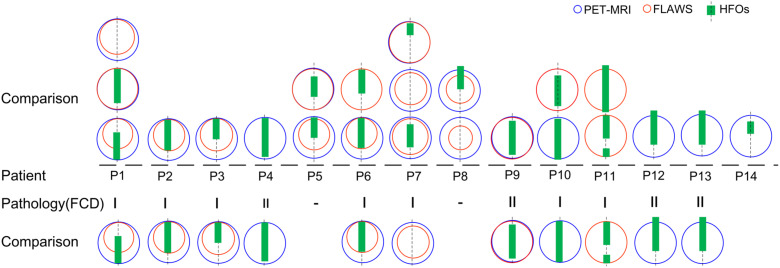
Comparison of HFO-fast ripple (FR) marking and lesions detected by neuroimaging. A comparison of the extent of areas marked using the three methods in 14 patients is shown (patient #15 was not shown as no FR was detected); blue and red circles represent PET-MRI and FLAWS, respectively. Green rectangles represent HFO-FR-marked areas of the implanted electrodes (symbolized by the black imaginary line). Lesions classified as FCD were further compared, and HFO-FR-marked areas even extend beyond the FCD lesion.

Interestingly, when we only focused on patients’ lesions verified to be FCD indicated by pathology, HFO-marked areas were more likely to be the margins or even extended beyond the lesion. This indicated that the perilesion of FCD may contribute to epileptic seizures (Figure 3).

### Case Studies

**FIGURE 4:**
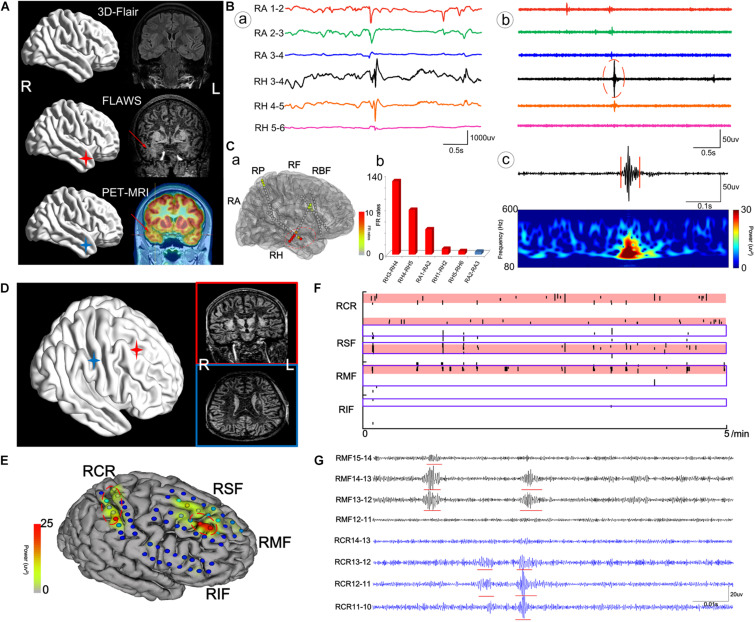
The cases of patient #2 **(A–C)** and patient #11 **(D–G)**. **(A)** The neuroimaging results for patient #2. **(B)** Raw data (a) and intracranial electroencephalogram (iEEG) signal after the bandpass filtering (200–500 Hz) (b) of some meaningful channels. (c) One FR detected using our detector. RA, right amygdala; RH, right hippocampus. **(C)** Results of HFOs shown on the model of the brain of patient #2 (a) and the rank in a descending order according to FR rates (b). The red color marks the suspicious channels indicated by the HFO threshold. RP, right parietal lobe; RF, right frontal lobe; RBF, right basis of frontal lobe. **(D)** The neuroimaging results for patient #11. **(E)** The FR rates shown on the brain model. RCR, right central areas; RSF, right superior frontal lobe; RMF, right mesial frontal lobe; RIF, right inferior frontal lobe. **(F)** The timing of all FRs detected in each channel with our automatic detector during a 5-min iEEG segment. The time and location of each FR are presented as points. The resected area is delineated by the blue line. The pink regions show the EZ confirmed using our quantified threshold. One area marked by HFOs had not been damaged. **(G)** The detectable FRs in different channels. The red arrows indicate the marked abnormal areas.

Patient #2 (Figures 4A–C), a 29-year-old male, had experienced tonic–clonic seizures for 24 years, but his MRI and 3D-FLAIR images revealed no abnormality. PET and MRI fusion results revealed hypometabolism of the right temporal lobe and right hippocampus, and FLAWS results also showed a lesion in the same area. After automatically detecting HFOs, the top 72% of the channel distribution area of HFOs covered the right hippocampus and amygdala, consistent with the PET-MRI and FLAWS results. Finally, the right hippocampus, right temporal lobe, and the right basis frontalis (little abnormal discharge) of the patient were removed, and the patient was seizure-free after 1 year of follow-up.

Patient #11 (Figures 4D–F), a 20-year-old male, had experienced physical convulsions or tonic–clonic seizures for 17 years. The MRI was normal. FLAWS indicated that the right frontal lobe and areas around posterior central sulcus were abnormal, as also detected by HFO analysis. However, due to the ignorance in the importance of using FLAWS and HFOs, and with the purpose of protecting the eloquent cortex, only the right frontal lobe of the patient had been removed, and the central area was not treated. As a result, the patient still had seizures after the surgery.

**FIGURE 5:**
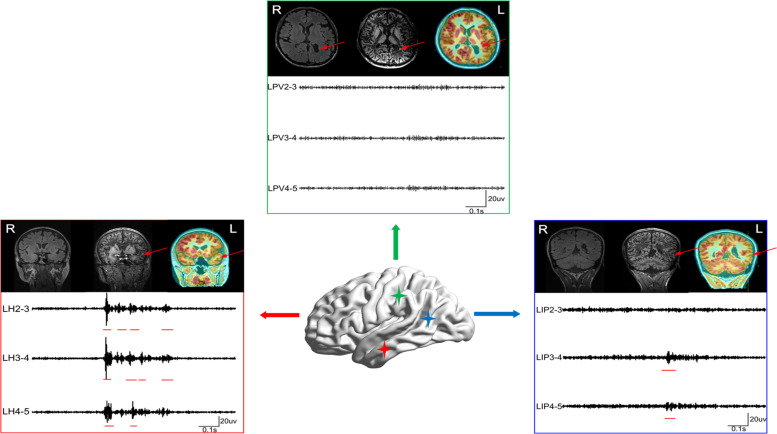
The case of patient #1. Patient #1 was suspected to have gray matter heterotopia in the left medial parietal lobe (near ventricle) based on the MRI result, as the heterotopia was obvious both in PET-MRI and FLAWS. According to the HFO analysis, channels located in this region exhibited greatly fewer FRs than channels in left hippocampus and left posterior temporal region. The three boxes represent three lesions marked using neuroimaging (red arrows) and results of FRs (red lines). LPV, left periventricle; LH, left hippocampus; LIP, left inferior frontal gyrus. The red arrows indicate the marked abnormal areas.

In addition, we must note that not all lesions are associated with EZs, such as in nodular heterotopia (NH). In this study, patient #1 (Figure 5), a 23-year-old man, had been suspected to have gray matter heterotopia in the left medial parietal lobe (near the ventricle), which was obvious both on PET-MRI and FLAWS images. According to the HFO analysis, channels located in this area were not the EZ. This heterotopic area was not removed. However, the patient was still seizure-free 1 year after the surgery.

## Discussion

For patients with drug-resistant epilepsy, removal of the EZ provides the best opportunity to eliminate seizures. However, the choice of methods that will accurately locate the EZ in a preoperative evaluation is a challenging problem that determines the success of the operation. This is particularly true for the group of patients with intractable partial epilepsy who do not have an unequivocal, single, focal epileptogenic lesion on MRI (Knowlton, 2006). Multimodal methods formulated to detect the EZ have been published before, but have often focused on common imaging tests such as single-photon emission CT (SPECT), FDG-PET, diffusion tensor imaging (DTI), and so on (Pustina et al., 2015; Perry et al., 2017). When analyzing EEG results, the electrical signals analyzed usually were spikes or those at a low-frequency band. Recently, several new imaging methods have been developed to detect these MRI-invisible lesions, including PET-MRI, FLAWS, and so on. The aim of this study was to compare the accuracy of these methods to localize the EZ. To our knowledge, this clinical investigation is the first to focus on an automated analysis of HFOs, PET-MRI, and FLAWS as a multimodal method to locate the EZ.

As one of the non-invasive methods, FDG-PET is the most established functional imaging modality used to evaluate patients with epilepsy. Presurgical FDG-PET scans of patients with epilepsy are typically performed with the goal of detecting focal areas of decreased metabolism that are presumed to reflect focal functional disturbances in cerebral activity associated with EZs (Knowlton, 2006). With advances in technology, the combination of FDG-PET and MRI exams has been shown to increase the detection of the seizure focus and to improve the outcomes of epilepsy surgery to a greater extent than with either modality alone (Lee and Salamon, 2009). In particular, new hybrid PET/MR scanners potentially combine the superior soft tissue contrast of MRI and the metabolic characterization of FDG-PET in a single exam, without the need for additional ionizing radiation inherent to PET/CT systems. According to numerous studies, PET-MRI enhances the non-invasive detection of EZ, particularly in patients with subtle lesions (Salamon et al., 2008; Chassoux et al., 2010), as well as in patients with temporal lobe epilepsy (TLE) (Lopinto-Khoury et al., 2012). When the patients’ cortical lesions are more clearly identified, this technique enables a more precise localization of the borders of the EZ. However, some critical issues should be carefully addressed before drawing a diagnostic conclusion from a PET/MRI fusion image (Hu et al., 2018). First, the reduction in glucose uptake may be a physiological phenomenon that is irrelevant to epileptogenic seizures. Second, the hypometabolic areas usually appear to be larger than the real EZ because FDG uptake includes a larger area. Last, since some small FCD lesions are located at the bottom of a deep sulcus (Besson et al., 2008), PET-MRI may be insensitive to detect mild hypometabolic changes and, thus, may miss some meaningful areas that generate seizures.

Abnormalities such as FCD are often subtle, and thus, MR sequences that provide optimal contrast between normal and abnormal tissues to detect minor lesions in the cortex are urgently needed. FLAWS is a new method that clearly shows the gray matter by selectively suppressing signals from the CSF and normal white matter. The feature of acquiring two image datasets with different inversion times within the same sequence and head geometry gives the opportunity to create calculated images harnessing specific tissue contrasts, which might be helpful in segmentation algorithms and clinical diagnosis (Tanner et al., 2012). This method has the potential to detect subtle lesions that are amenable to visualization using a conventional MRI. As shown in the study by Chen et al., when the reviewers were blinded to the location of the resected regions, the FCD detection rate of the FLAWS sequence was significantly higher than that of the conventional sequences (*p* = 0) (Chen et al., 2018). However, as proved in our study, this method is only sensitive to lesions located in the cerebral cortex, but not to abnormalities in the hippocampus and other deeper brain structures.

For suspicious lesions detected using neuroimaging, clinicians usually implant intracranial electrodes into the suspicious areas and further evaluate the location of the EZ based on intracranial EEG signals. HFOs have been increasingly recognized as a promising biomarker for locating the EZ, especially for FRs (Wu et al., 2010; Zijlmans et al., 2012; Fedele et al., 2016; Fedele et al., 2017). Jiang et al. (2018) have suggested that the quantitative threshold for FR distribution to delineate the EZ is set to continuously remove at least 72% of the channels from the highest ranking. In this experiment, we used this threshold to locate the EZ. However, not every patient had FRs detected, like the patient #15, in which case ripple results would be referred.

The spatial resolution of HFOs to locate the EZ is significantly more accurate than that of PET-MRI and FLAWS, but our HFO analysis was based on the electrical signals of the intracranial electrodes, making it an invasive examination. The accuracy of localizing the epileptogenic area depended on whether the area in which the intracranial electrode was implanted had covered the entire EZ.

Since each examination has its advantages and disadvantages, a multimodal comparison that combines PET-MRI, FLAWS, and HFOs is strongly recommended to help ensure maximum precision in localizing the EZ. This may overcome the intrinsic limitations of individual modalities, particularly for patients with a negative MRI and suspicious cortical lesions. First, using non-invasive methods such PET-MRI and FLAWS to detect metabolic changes and structural abnormalities, respectively, clinicians will be able to identify the suspicious areas related to clinical seizure semiology. Then, clinicians can implant electrodes in these areas to monitor electrical signals. According to the HFO results obtained from the intracranial electrodes, clinicians can further pinpoint the optimal boundaries of the EZ and discover some subtle lesions that might be missed by neuroimaging. Finally, overlapping areas identified in the multimodal comparison can be surgically resected.

In our study, the percentage of seizure-free was only 33.33% (5/15), lower than the effective rate reported before. This was because only patients having trouble locating the EZs would undergo these new examinations, and this research was a retrospective study, which meant that the surgery plans were not based on the results. In the future, when the results from multimodal comparing are put into practice, patients will benefit more.

In this study, when we focused on patients’ lesions that were verified to be FCD, the HFO-marked area did not completely match the FCD areas. In actuality, the areas of FCD may display different degrees of epileptogenicity, ranging from electrographic silence to interictal epileptic discharges, and an initial involvement in seizure generation. The high variability in the epileptogenicity of these lesions may explain why some patients with FCD become seizure-free, despite an incomplete resection of the lesion (Fauser et al., 2009). We found that HFO-marked areas were more likely to be the margins of the dysplastic cortex and that perilesions of FCD may also be epileptic. This result is consistent with a previous study by Ferrari-Marinho et al. (2015). The occurrence of a high rate of HFOs reflects the epileptic disease activity of the underlying lesion and can be used to evaluate the different grades of epileptogenicity. This tool can help clinicians define the real extent of the EZ beyond the visible lesion observed using neuroimaging.

Compared with FCD, nodular heterotopia appears to be more complex. Nodular heterotopia results from a migration disorder characterized by the ectopic position of neurons that are frequently located along the ventricular walls (periventricular nodular heterotopia, PNH) or in the deep white matter in the form of nodules (subcortical heterotopia) (Barkovich et al., 2012). Most patients with NH suffer from drug-resistant epilepsy, but some do not show seizures (Raymond et al., 1994; Battaglia et al., 1997). In (Jacobs et al., 2009), reported that patients with NH often had SOZ areas outside the lesion, and FRs and spikes were more closely related to SOZ than to the lesion (Jacobs et al., 2009). In (Pizzo et al., 2017), found that only 6% of seizures were purely heterotopic (Pizzo et al., 2017). In (Scholly et al., 2018), recommended a combination of HFO and spike analysis to locate the EZ of PNH (Scholly et al., 2018). Our findings from patient #1 are consistent with these studies and highlight the necessity of combining results from HFO analysis and neuroimaging to analyze EZs.

A limitation of our study is that our study is an exploratory experiment. Consequently, the number of included patients is small. In the future, we will obtain data from more patients to verify the possibility of using the multimodal method to accurately locate the EZ.

## Data Availability Statement

The raw data supporting the conclusions of this article will be made available by the authors, without undue reservation.

## Ethics Statement

The studies involving human participants were reviewed and approved by the Ethics Committee of Xuanwu Hospital. Written informed consent to participate in this study was provided by the participants’ legal guardian/next of kin.

## Author Contributions

XL, TY, ZR, XW, and XC analyzed the data. XL, HZ, and XMY wrote and revised the manuscript. All authors contributed to the study and approved the submitted version.

## Conflict of Interest

XZ was employed by the company Siemens Healthcare. The remaining authors declare that the research was conducted in the absence of any commercial or financial relationships that could be construed as a potential conflict of interest.

## Footnotes

1 https://www.fil.ion.ucl.ac.uk/spm/software/spm12/

## Supplementary Material

Supplementary Table 1 Comparing the new and traditional methods’ results. Lmp, left medial parietal; Lh, left hippocampus; Lt, left temporal; Lpt, left posterior temporal; Rhmei, Right Hemisphere; Racc, right anterior cingulate cortex; Rmcc, right middle cingulate cortex; Rbf, right basis frontalis; Rt, right temporal; Ri, right insula; Rh, right hippocampus; Ra, right amygdala; Rp, right parietal; Rsp, right superior parietal; Rip, right inferior parietal; Lp, left parietal; Lo, left occipital; Rpt, right posterior temporal; Rf, right frontal; Rcr, right central region; Rcs, Right central sulcus; BiF, bilateral frontal; BiT, bilateral temporal. #, No FR was detected, the results were from ripples.
