# Supplementary material for: Localization of the Epileptogenic Zone by Multimodal Neuroimaging and High-Frequency Oscillation
Source: Front Hum Neurosci. 2021 Jun 8;15:677840. doi: 10.3389/fnhum.2021.677840 (PMC8217465; doi:10.3389/fnhum.2021.677840)
Supplement: Supplementary Table 1 — Comparing the new and traditional methods’ results. Lmp, left medial parietal; Lh, left hippocampus; Lt, left temporal; Lpt, left posterior temporal; Rhmei, Right Hemisphere; Racc, right anterior cingulate cortex; Rmcc, right middle cingulate cortex; Rbf, right basis frontalis; Rt, right temporal; Ri, right insula; Rh, right hippocampus; Ra, right amygdala; Rp, right parietal; Rsp, right superior parietal; Rip, right inferior parietal; Lp, left parietal; Lo, left occipital; Rpt, right posterior temporal; Rf, right frontal; Rcr, right central region; Rcs, Right central sulcus; BiF, bilateral frontal; BiT, bilateral temporal. #, No FR was detected, the results were from ripples. [file Table_1.DOCX]

**Supplementary Table 1 Comparing the new and traditional methods’ results**

| Patient | MRI | scalp EEG | iEEG | PET-MRI | FLAWS | HFOs |
| --- | --- | --- | --- | --- | --- | --- |
| 1 | LMP | BiF,LT,LP,LO | LH | LT, LH, LPT, LMP | LT, LH, LPT, LMP | LH, LPT |
| 2 | normal | RHemi | RACC, RBF | RT, RH | RT, RH | RH, RA |
| 3 | RH | RP | RP | RSP | RP | RP, RIP |
| 4 | LH | LPT, LP, LO | RPT | RPT | normal | RPT, LH |
| 5 | normal | RCR | RCS | RCS | RCS | RP,RCS |
| 6 | normal | RHemi | RF | RT, RP | RF, RCR | RF, RCR |
| 7 | BiF | BiT | LF | LF,LOF, LTP, LH | LF, LOF, LT, LO, RO | LF, LH, RA |
| 8 | RH | normal | RT, RI | RF,RT,RI | RT, RI | RT |
| 9 | normal | RF | RF | RF | RF | RF |
| 10 | LTP | LT | LT | LP,LI | LT | LO,LT,LI |
| 11 | normal | RHemi | RF | / | RF, RCR | RF, RCR |
| 12 | LT | LPT | LF | LF | / | LF |
| 13 | normal | LF | LF | LF | / | LF |
| 14 | normal | RT,RCR | RF | RF | / | RF,RMCC |
| 15 | RF | RF | RF | RF | / | RF(#) |

LMP: left medial parietal; LH: left hippocampus; LT: left temporal; LPT: left posterior temporal; RHmei: Right Hemisphere; RACC: right anterior cingulate cortex; RMCC: right middle cingulate cortex; RBF: right basis frontalis; RT: right temporal; RI: right insula; RH: right hippocampus; RA: right amygdala; RP: right parietal; RSP: right superior parietal; RIP: right inferior parietal; LP: left parietal; LO: left occipital; RPT: right posterior temporal; RF: right frontal; RCR: right central region; RCS: Right central sulcus; BiF: bilateral frontal; BiT: bilateral temporal.#: No FR was detected, the results were from ripples.
